# A Novel Superabsorbent Polymer from Crosslinked Carboxymethyl Tragacanth Gum with Glutaraldehyde: Synthesis, Characterization, and Swelling Properties

**DOI:** 10.1155/2021/5008833

**Published:** 2021-11-20

**Authors:** Yahya Bachra, Ayoub Grouli, Fouad Damiri, Mohammed Talbi, Mohammed Berrada

**Affiliations:** ^1^Laboratory of Biomolecules and Organic Synthesis (BIOSYNTHO), Faculty of Sciences Ben M'Sick, Department of Chemistry, University Hassan II of Casablanca, Casablanca, Morocco; ^2^Innovations and Technologies Platform (PInTech), University Hassan II of Casablanca, Casablanca, Morocco; ^3^Laboratory of Analytical Chemistry and Physical Chemistry of Materials, Faculty of Sciences Ben M'Sick, University Hassan II, Casablanca, Morocco

## Abstract

Nowadays, current global environmental problems include measures to eliminate or reduce the negative impact of chemicals from petroleum sources and, therefore, the use of materials from natural resources is increasingly recommended. In this context, natural-based superabsorbent polymers derived from polypeptides and polysaccharides have undergone chemical and biochemical modifications to improve their ability to absorb and retain large amounts of liquids. In the present paper, a new process has been used to overcome the side effects of radical polymerization in the manufacture of conventional polyacrylate superabsorbents (SAPs). Tragacanth gum (TG) was selected to prepare a new superabsorbent material (CMTG-GA) based on carboxymethyl tragacanth (CMTG) crosslinked with glutaraldehyde (GA). The characterization of the polymer was carried out by FTIR, TGA, XRD, and SEM. The effect of the amount of crosslinking agent and the pH on the water absorption capacity was also examined. Subsequently, swelling studies were performed using free swelling capacity (FSC) and centrifuge retention capacity (CRC) techniques in distilled water, tap water, and saline solution. The results showed that the CRC of the new material is not less than 42.1 g/g, which was observed for a ratio of 20% by weight of GA to CMTG. Likewise, the maximum absorption results were 43.9 and 32.14 g/g, respectively, for FSC and CRC at pH 8.0. In addition, a comparison of the swelling capacities of the synthesized product with a commercial SAP extracted from a baby diaper, well known in the Moroccan market, showed that the performances were very similar.

## 1. Introduction

It is well known that superabsorbent polymers (SAPs) are highly consumable products in everyday life, and the demand for products prepared with these polymers is very high (e.g., baby diapers, sanitary napkins, and incontinence articles) [1]. The products currently on the market are almost entirely synthetic materials derived from petroleum, which have negative effects on the environment on the one hand and, on the other hand, their price is relatively high and depends on the market price of petroleum [2]. The step of investing in the research and development of alternatives is therefore very promising.

SAPs have high water absorption due to the increase in specific surface area, resulting in greater exposure of water to hydrophilic groups in the polymer backbone. Several parameters influence the swelling process, which contributes to the final swelling capacity. These include hydrogen bonding, the effect of crosslinking, and the content of hydrophilic groups in the polymer backbone such as carboxylate ions in the case of anionic polymers. SAPs are mainly classified as synthetic based on petrochemicals and natural based on biopolymers. The majority of SAPs used in commercial applications today are of synthetic origin. They are made from nonrenewable petrochemical resources and in particular acrylate and acrylamide-based monomers [3, 4]. Petrochemical-based SAPs are most often technically prepared by radical polymerization of vinyl monomers in the presence of multifunctional crosslinking agents [5, 6]. It is clear that synthetic polymers suffer from such disadvantages as environmental pollution and toxicity of the synthesis process, side effects of traces of initiator and residual acrylic monomers, poor consumer compliance, and sometimes high price. Their synthesis requires a long development time compared with natural polymers [7–9]. However, natural SAPs are prepared from polypeptides or polysaccharides such as cellulose, starch, agarose, chitosan, alginates, carrageenan, and their derivatives. These polymers lend themselves easily to chemical and biochemical synthesis modifications to improve their ecological characteristics [10–12].

**Figure 1:**
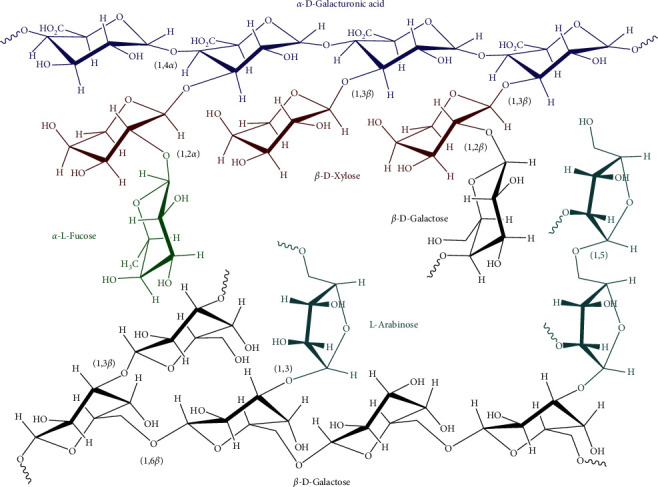
Chemical structure of TG, adapted from [20].

Natural gums or polysaccharide gums are hydrophilic polysaccharides obtained from renewable sources such as microbial, marine, or plant sources [13]. Depending on the source, they are classified as microbial gums, vegetable exudate gums, or seed gums, thus representing one of the most abundant raw materials in nature [14]. Natural polymers find many applications in a wide range of fields because they are readily available, relatively inexpensive, nontoxic, and environmentally friendly [15]. In addition, they also have extensive industrial applications due to their ability to hydrate in cold or hot water, either by stabilizing emulsion systems or by gel formation [16]. Tragacanth gum (TG), a very complex heterogeneous anionic polysaccharide, is formed as a dried exudate from the stems and branches of *Astragalus gummifer* and other Asian species of *Astragalus*, such as *Astragalus gossypinus* and *Astragalus microcephalus*. It is produced by the disintegration of the plant via a process called gummosis [17]. The exudate solidifies in the form of flakes or coiled ribbons and can be collected after a few weeks. With a high molecular weight of up to 850 kDa, TG is found in Turkey, Afghanistan, India, and Iran [18, 19]. Analysis of the composition of TG identified the presence of residues of arabinose, glucose, xylose, galactose, rhamnose, fucose, and galacturonic acid in the chemical structure of TG as shown in Figure 1. Due to geographic and seasonal variations, the ratio of each sugar varies considerably in the gums obtained from different species of *Astragalu*s [21].

TG consists of two main fractions called tragacanthin and bassorin, which are both insoluble in alcohol [22]. However, tragacanthin is a highly branched, water-soluble mixture of D-galacturonic acid and other sugar residues [23], whereas bassorin, an almost linear arabinogalactan, is a water-swelling polymer that appears almost neutral and contains a high ratio of L-fucose, D-xylose, L-arabinose, D-galactose, and mainly the methyl D-galacturonic ester instead of the acidic units in its structure [24–26]. Actually, commercial TGs have large differences in chemical composition, including sugar composition, methoxy content, and the relative proportion of soluble and insoluble components [19]. TG is a medically important polysaccharide that has been approved by the FDA [14]. It is also nontoxic, stable over a wide pH range, biocompatible, and safe for oral intake according to REACH regulations [27]; the fields of application of TG are therefore very wide. As a biomaterial, TG has been applied in industrial settings, such as in food packaging [28], the processing of bioproducts as an additive [29], cosmetics as an emulsifying and suspending agent in a variety of pharmaceutical formulations [30], for environmental purposes of purification of water polluted with dyes and toxic heavy metal ions [31, 32], nanofibers and textiles [33], in the green synthesis of reducing agents and stabilizers [34], for antimicrobial applications [35], biomolecules and drug delivery [36], wound healing [37], bone tissue engineering [38], immobilization and cell growth matrix [39], as well as the regeneration of damaged peripheral nerves [40].

Although pure tragacanth gum has a degree of absorption capacity, it still raises questions. By mixing TG with water, it can swell as the insoluble fraction, bassorin (60–70%), swells to form a gel [38]. The acidic and ionic units in the chemical structure of gums are responsible for their different hygroscopic properties. Studies on the water absorption properties of various gums at temperatures between 20 and 65°C have shown that TG has higher water absorption than guar gum and locust bean gum [41]. Therefore, it can be considered an absorbent material but not yet a superabsorbent material. Recently, some researchers have developed SAPs based on TG. For example, a superabsorbent copolymer synthesized by graft polymerization of acrylic acid onto carboxymethyl tragacanth gum [42], a superabsorbent composed of TG and polyethylene oxide (PEO) obtained by gamma radiation [43], as well as a bioplastic absorbent material produced by a mixture of TG and egg white [44]. As indicated above, the use of biopolymers as superabsorbent materials requires some chemical modification, in particular increasing the number of anionic parts in the saccharide backbone. The chemical process of carboxymethylation of biopolymers involves the reaction of hydroxyl groups with saccharide units. It has been reported as one of the methods of grafting carboxylate groups into the matrix of biopolymers such as cellulose [45], starch [46], chitosan [47], guar gum [48], tragacanth gum [42], and tamarind gum [49]. Unlike conventional crosslinking techniques, acetalization with dialdehydes has been reported as an environmentally friendly method to avoid radical polymerization, based on acrylic monomers and MBA (N,N'-methylenebismethacrylamide). Thus, glutaraldehyde (GA), a dialdehyde generally used for protein binding and sterilization, can also be induced to form acetal groups by reaction with hydroxyl groups [50–52].

Nowadays, considering the turbulent economic conditions that the world is facing due to the consequences of the COVID-19 pandemic that is creating a global war on the price of petroleum and its derivatives, the biggest challenge in the field of biodegradable SAPs is to synthesize or manufacture superabsorbent materials based on fully biodegradable polymers that would absorb and retain water rapidly and reversibly [2]. For these reasons, our approach to the development of SAPs is to synthesize an environmentally friendly superabsorbent material based on a fully biodegradable polymer (tragacanth gum) that would absorb and retain water rapidly and reversibly while being competitive with synthetic SAPs on the market in terms of absorption capabilities under specific conditions and market price. Therefore, the approach will be carried out through a carboxymethylation reaction followed by acetalization crosslinking to avoid the side effects of radical polymerization as described above.

In the present study, we have synthesized a new superabsorbent polymer (CMTG-GA) by first preparing carboxymethyl tragacanth (CMTG) via the process of carboxymethylation and then synthesizing the crosslinked carboxymethyl polymer using glutaraldehyde (GA). The characterization of the polymers was carried out by FTIR, TGA, XRD, and SEM. The swelling performance was studied via the free swelling capacity (FSC) and the centrifuge retention capacity (CRC). The effect of the synthesis parameters on the water absorption capacity was studied, in particular the amount of crosslinking agent and the pH. Finally, a comparison between the performance of the synthesized product and the swelling capacity of commercial products was carried out.

## 2. Materials and Methods

### 2.1. Materials

Tragacanth gum (TG) was purchased from the local market in Casablanca, Morocco. The gum was crushed and used without further purification. Glutaraldehyde (GA, 25% aqueous solution) was of grade II and was used as a crosslinking agent, which was purchased from Sigma-Aldrich (USA). Chloroacetic acid, isopropyl alcohol, acetic acid, hydrochloric acid, and sodium hydroxide were of analytical grade (extra pure) and were used as supplied by Sigma-Aldrich (USA) without further purification. Acetone and methanol were of analytical standard grade (99.7% purity) from VWR Chemicals (USA). To adjust the pH, acidic and basic solutions were prepared by diluting solutions of hydrochloric acid (pH 1.0) and sodium hydroxide (pH 13.0) with distilled water. For the study of the swelling behavior of SAPs, saline solution was prepared as 0.9% (w/v) NaCl (sodium chloride), and the sodium chloride was of analytical grade (99% purity) from Loba Chemie (India). In addition, the total hardness of the tap water was between 100 and 140 ppm calcium carbonate. Finally, to compare the performance of the SAP product, a water-absorbent material from the commercial diaper, “Pampers” (Procter & Gamble Ltd.), later called commercial SAP, was used as a control.

### 2.2. Experimental

#### 2.2.1. Synthesis of CMTG

**Figure 2:**
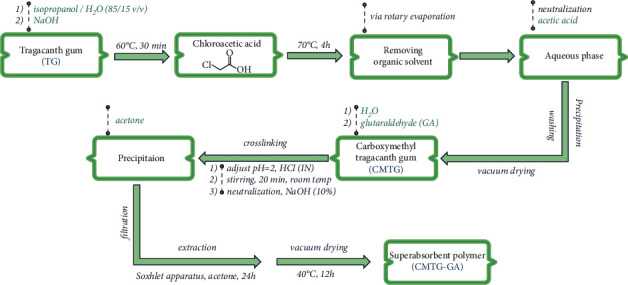
Schematic representation of the stepwise synthesis process of CMTG-GA.

Carboxymethyl tragacanth, CMTG, was prepared under conditions similar to those reported elsewhere [42, 46]. First, in a 100 ml three-necked round-bottom flask equipped with a reflux condenser and a magnetic bar, 1 g of TG was uniformly dispersed in 25 ml of an isopropanol/H_2_O (85/15 v/v) solvent mixture, and then 1.2 g of NaOH was further added to the reaction mixture. The mixture was heated to 60°C. In the meantime, 1.5 g of chloroacetic acid was gradually added after 30 minutes. The reaction was allowed to continue at 70°C for an additional 4 hours, and then the round-bottom flask was allowed to cool to room temperature. At this point, using rotary evaporation, the organic solvent was removed under reduced pressure and the aqueous phase was neutralized with acetic acid. Then, 30 ml of cold methanol (2°C–4°C) was added to the reaction mixture as a nonsolvent and the precipitate was collected, washed several times with excess methanol, and dried in a vacuum (Figure 2). The yield was about 60%.

#### 2.2.2. Synthesis of CMTG-GA

In a round-bottom flask equipped with a magnetic stir bar, 0.5 g of the obtained CMTG was dissolved in 60 ml of distilled water and then the desired weight ratios of GA (Table 1) were added to the flask. HCl (1N) was used to adjust the pH of the mixture to 2 and then stirred continuously for 20 minutes at room temperature. The mixture was then neutralized by adding a few droplets of NaOH solution (10%), and then the precipitation was completed by pouring the mixture into acetone and then filtered. Finally, in a Soxhlet apparatus using acetone, the filtered precipitate was extracted for 24 h. The extracted product was dried successively overnight under vacuum at 40°C, and then the product was further ground (Figure 2).

### 2.3. Characterization

#### 2.3.1. Fourier Transform Infrared Spectroscopy (FTIR)

Fourier transform infrared spectroscopy (FTIR) spectra of TG, CMTG, and CMTG-GA were recorded with the preparation of KBr pellets on a Bruker Tensor-27 spectrophotometer from Bruker Corporation (Germany). The infrared transmittance method was used, and all spectra averaged 32 scans from 4000 to 400 cm^−1^ at a resolution of 4 cm^−1^.

#### 2.3.2. Thermogravimetric Analysis (TGA)

Thermogravimetric analysis (TGA) was carried out on a Setsys Evolution 16/18 TGA/DTA instrument (SETARAM). The samples were subjected to a heating rate of 10°C/min in a heating range of 42 to 800°C.

#### 2.3.3. X-Ray Diffraction Analysis (XRD)

X-ray diffraction (XRD) patterns of TG, CMTG, and CMTG-GA were analyzed using Bruker D8-Advance X-ray powder diffractometer (Germany) with Nickel-filtered Cu-K*α* radiation (*λ* = 1.54056° A) at a voltage of 40 kV and current of 100 mA. The scattered radiation was detected in the angular range of 10–80° (2*θ*), with a step size of 0.01° (2*θ*).

#### 2.3.4. Scanning Electron Microscopy (SEM)

Morphological analysis of TG and CMTG-GA was performed by scanning electron microscopy (SEM) using a MiniSEM Hirox model SH-4000M.

### 2.4. Swelling Properties

To compare the synthesized products with the commercial products, the standards for evaluation of the absorption of superabsorbent polymers established and recommended by recognized international organizations were applied. Therefore, FSC and CRC were chosen as industrial-grade absorption tests [2, 53, 54].

#### 2.4.1. Free Swell Capacity (FSC)

The “free swelling capacity” is commonly referred to as the “teabag method.” It refers to the amount (g) of fluid absorbed per gram of composition. This technique is the most practical and rapid assessment of the absorption capacity of the absorbent sample [2]. The basic fluids are distilled water, tap water, and saline (0.9 wt% NaCl solution). The teabags were filled with (0.5 g ± 0.01) TG, CMTG-GA, and commercial SAP samples, evenly distributed in the teabag, soaked in distilled water, pure tap water, or saline solution for 30 minutes, removed to achieve equilibrium swelling, and let stand for 15 minutes to drain off excess solution, and then weighed. Empty teabags have also been prepared and go through the same steps to serve as controls. Three controls were prepared for each sample to be tested to determine the average absorption factor (F, g/g) of the blank teabag according to

(1) $$\begin{array}{c} F\left(\frac{g}{g}\right)\;=\frac{\left(T_{2}-T_{1}\right)}{T_{1}}, \end{array}$$

where *F*, *T*_2,_ and *T*_1_ are, respectively, the absorption factor of the teabag, the weight of the filtered empty teabag (control), and the weight of the empty dry teabag (control). Therefore, the free swelling capacity is calculated using

(2) $$\begin{array}{c} \text{FSC}\left(\frac{g}{g}\right)=\frac{\left(W_{2}-\left(W_{1}-\bar{F}\right)-W_{1}-W_{0}\right)}{W_{0}}, \end{array}$$

where *W*_2_ is the weight of the swollen sample, *W*_1_ is the weight of the empty dry teabag, *W*_0_ is the initial weight of the SAP sample, and ¯ $\bar{F}$ is the average teabag absorption factor (g/g). The FSC of each particle sample was determined by averaging the results of three samples tested.

#### 2.4.2. Centrifuge Retention Capacity (CRC)

This approach refers to the ability of TG, CMTG-GA, and commercial SAP samples to retain fluids after being saturated and centrifuged under controlled conditions. The resulting retention capacity was measured in grams of fluid retained per gram of sample weight (g/g). The samples to be tested were the same teabags (including controls) collected in the previous FSC test.

The teabags were placed in a centrifuge or spinner equipped with a basket rotor capable of subjecting the samples to a g-force of about 250 G applied to a mass placed on the inner wall of the basket. The teabags were placed on the internal wall of the basket, centrifuged at 250g (1400 rpm) for 3 minutes, and then removed and weighed. The amount of solution retained by the SAP sample, taking into account the solution retained by the teabag itself, was the centrifuge retention capacity (CRC) of the samples. However, the retention factor (*F*, g/g) of the teabag controls was also calculated using equation (1). The retention capacity of the centrifuge is then calculated using

(3) $$\begin{array}{c} \text{CRC}\left(\frac{g}{g}\right)=\frac{\left(W_{2}-\left(W_{1}-\bar{F}\right)-W_{1}-W_{0}\right)}{W_{0}}, \end{array}$$

where *W*_2_ is the weight of the centrifuged teabag (g), *W*_1_ is the weight of the empty and dry teabag (g), *W*_0_ is the initial weight of the SAP sample (g), and ¯ $\bar{F}$ is the average retention factor teabag (g/g). Three tests of each particle sample were tested, and the results were averaged to determine the CRC.

## 3. Results and Discussion

### 3.1. Synthesis of CMTG-GA

Tragacanth gum has a hydrophilic behavior and can swell in the presence of a dissolution medium. As is well known, the swelling capacity in water can be improved by increasing the hydrophilic parts and the hydrogen bonds of the polysaccharide backbone. According to this hypothesis, this synthesis aimed to increase the swelling capacity of TG to convert it into a superabsorbent polymer. The process consisted of increasing the number of anionic groups on the glycosidic chains and then partially crosslinking these chains by an organic synthesis instead of radical polymerization based on acrylic or acrylamide monomers.

**Figure 3:**
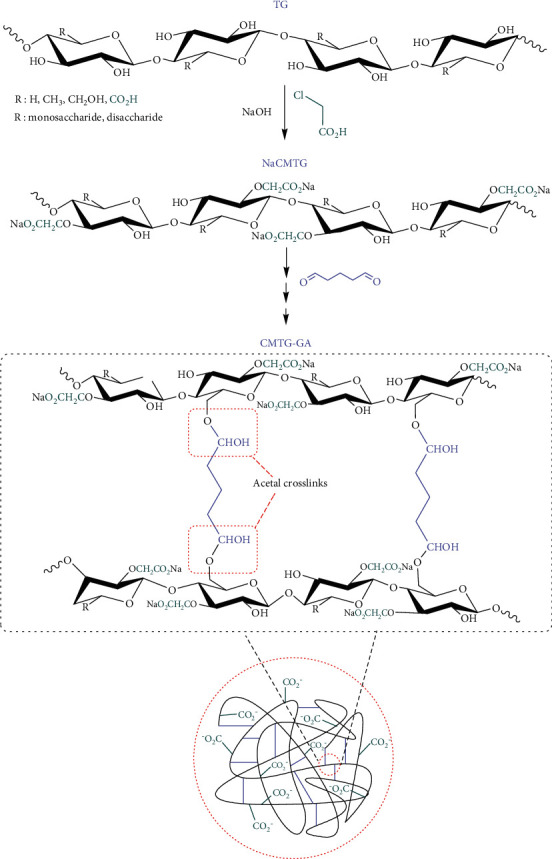
The plausible proposed mechanism for the synthesis of the CMTG-GA.

CMTG-GA hydrogels with different weight ratios of GA to CMTG were synthesized in two steps. First, CMTG was synthesized from the reaction between TG and chloroacetic acid in NaOH, which acted as an activator. This reaction is illustrated in Figure 3.

These active sites reacted with carbon that has the leaving group of chloroacetic acid to form covalent bonds. It should be noted that the NaOH ratio is very important in the processes as sodium glycolate can be formed by a secondary reaction as a by-product if NaOH has been used excessively.

The prepared CMTG is a multifunctional polymer containing a large number of reactive carboxylate groups that convert it into a hydrophilic material capable of attracting water and similar polar molecules or groups. As well, it is also capable of forming new bonds by reacting with reactive groups such as hydroxyl groups of polysaccharides distributed on glycosidic chains. However, it must be partially crosslinked to upgrade it to the superabsorbent level. Glutaraldehyde (GA) is a bifunctional molecule that has two aldehyde groups at the end of the carbon chain. Using most of its functionality, it has the potential to form a random three-dimensional network structure by incorporating a suitable counterpart through a hemiacetalization reaction without any external catalyst. Obviously, GA has been widely used for the crosslinking of polymers containing hydroxyl groups. Thus, it can also crosslink carboxymethylated tragacanth gum to form a hydrogel.

In the second step, CMTG-GA hydrogels were synthesized by crosslinking the CMTG polysaccharide backbones using GA as the crosslinking agent. The plausible mechanism of synthesis of the superabsorbent polymer CMTG-GA is given in Figure 3. The aldehyde groups of the GA reacted with the hydroxyl groups of the CMTG to form hemiacetal crosslinks. Finally, the superabsorbent polymer with a three-dimensional network was obtained. The formation of hemiacetal and acetal bonds between CMTG and glutaraldehyde during crosslinking is often unstable and reverts to its parent components. This can, therefore, lead to the formation of a semi-IPN hydrogel. From an environmental point of view, this instability can be considered an influential process that facilitates the mechanisms of biodegradation of the superabsorbent after use.

### 3.2. FTIR Analysis

**Figure 4:**
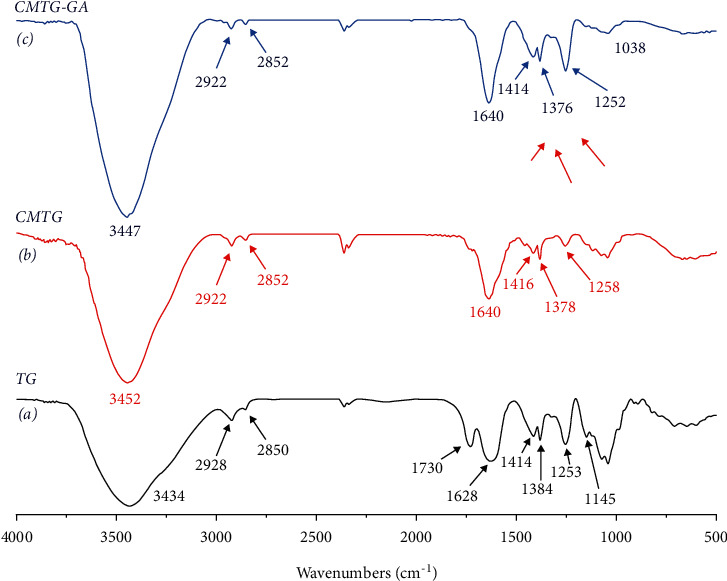
FTIR spectra: TG (a), CMTG (b), and CMTG-GA (c).

The FTIR spectra of TG (a), CMTG (b), and CMTG-GA (c) are shown in Figure 4.

Figure 4 illustrates the characteristic bands of TG. The broad bands at 3434 cm^−1^ correspond to the stretching vibration of the hydrogen bond due to O—H groups, and the bands at 2928 and 2850 cm^−1^ are related to asymmetrical and symmetrical stretching vibrations of C—H groups, respectively. The absorption band at 1730 cm^−1^ is assigned to acidic and ester carbonyl groups C = = O; besides, the presence of the carboxylate groups of D-galacturonic acid is confirmed by the presence of the bands at 1658 cm^−1^. Besides, the C—OH deformation vibration with the contribution of O—C—O symmetrical stretching vibration of carboxylate groups is confirmed by the presence of the bands at 1414 and 1384 cm^−1^. The band at 1145 cm^−1^ corresponds to the C—O—C asymmetric stretching vibration in the glycosidic groups of the polysaccharides.

The FTIR spectrum of CMTG is presented in Figure 4, and the absorption bands at 3452, 2922, 2852, and 1378 cm^−1^ are assigned to the same vibrations of TG as described above. The wide absorption band appeared at 1640 cm^−1^ corresponded to the result of extension vibration of the carboxylate groups, which overlapped with acidic and ester carbonyl C = = O groups of TG monomers presented in the CMTG structure, thus confirming the addition of carboxylate groups by the carboxymethylation process. Finally, Figure 4 represents the FTIR spectrum of CMTG-GA. An apparent band at 1414 cm^−1^ is related to the –CH_2_ bending vibration of the glutaraldehyde parts. The band at approximately 1252 cm^−1^ is attributed to H_2_C—O—C due to the acetal crosslinks. Furthermore, the increase in the intensity of this band, compared with the band at 1258 cm^−1^ indicated in the CMTG spectrum, confirms the partial crosslinking of the CMTG chains by glutaraldehyde. Therefore, these results are evidence that the crosslinked polymer has been formed.

### 3.3. Thermal Stability Analysis

Generally, the decomposition of polysaccharides consists of four main stages: firstly, the desorption of physically absorbed water; the excretion of bounded water by dehydration reactions; chain depolymerization leading to monomer parts; and finally the creation of a polynuclear aromatic hydrocarbon [55].

**Figure 5:**
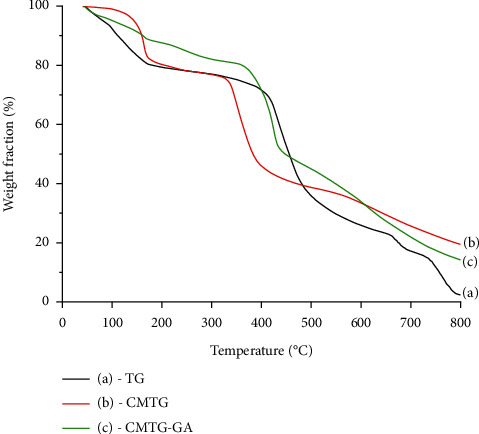
TGA curves of TG (a), CMTG (b), and CMTG-GA (c).

The TGA curves of TG (a), CMTG (b), and CMTG-GA (c) are illustrated in Figure 5. The curve of the neat tragacanth gum (Figure 5(a)) shows that the initial weight loss of about 18% at 155°C is related to the absorbed moisture. The second loss was a two-step weight loss of about 21 and 59% at 165–405 and 450–730°C, respectively. These weight loss steps correspond to the decomposition of the highly branched heterogeneous structure of TG. The residue is 2% at 800°C. The CMTG thermal curve (Figure 5(b)) shows three main weight losses. The initial weight loss step at 152°C, about 18%, is assigned to the moisture loss. The second step at 320–430°C was about 37%, corresponding to the ring decomposition of saccharide units and the rupture of C-O-C glycosidic bonds in polymeric chains. The third weight loss was about 26% at 450–800°C, corresponding to the release of CO_2_ from the polymer backbone and the breakage of CMTG backbones [56, 57]. Finally, about 19% is the residue at 800°C.

However, CMTG-GA shows four weight losses with a residue of 14% (Figure 5(c)). The initial weight loss, which is about 13% at 50–145°C, is related to the dehydration of the remaining moisture during the polymer formation. At 175–358°C, the second weight loss was about 20% and corresponds to the debut of decomposition of the polymer network. The third weight loss was about 14% at 360–440°C, while the fourth weight loss was about 38% at 450–800°C. These latter weight losses correspond to the decomposition mechanism of the polymer. Indeed, this mechanism is initiated by the decomposition of the saccharide units followed by the decomposition of the three-dimensional matrix through the rupture of the acetal links and the rupture of the C-O-C glycosidic bonds.

In summary, the CMTG-GA polymer exhibits superior thermal stability compared to TG and CMTG up to 600°C. For example, at 500°C, a weight loss of 55% is observed on the CMTG-GA curve, compared to weight losses of 62% and 65% for CMTG and TG, respectively. This stability is due to the formation of a dense three-dimensional network crosslinked by the hemiacetalization process. At higher temperatures, however, it has a lower thermal stability than CMTG. This is due to the rupture of the hemiacetal crosslinks leading to the degradation of the three-dimensional network. Consequently, the thermal properties of TG, CMTG, and CMTG-GA are greatly influenced by polymer chain crosslinking. Furthermore, the difference regarding the thermal behavior of the three curves also confirms the formation of the superabsorbent polymer. Similar results were reported [14].

### 3.4. X-Ray Diffraction Analysis

**Figure 6:**
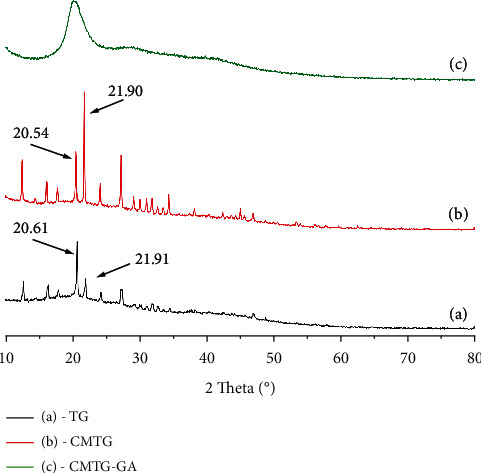
XRD patterns of TG (a), CMTG (b), and CMTG-GA (c).

The XRD patterns of TG (a), CMTG (b), and CMTG-GA (c) are shown in Figure 6.

From Figure 6(a), the XRD peaks at 2*θ* = 20.61° and 2*θ* = 21.91° were found to correspond to tragacanth gum (TG), indeed, it is evident that original tragacanth gum (TG) has slight crystallinity. A similar appearance has been reported for tragacanth gum in the literature [14, 58]. However, for CMTG, the diffraction peaks were found to shift to 20.54° and 21.90° (Figure 6(b)). It seems that after the carboxymethylation reaction, a distinct reduction in crystallinity occurs (Figure 6(b)). This loss in crystallinity could be attributed to the effect of the replacement of the hydroxyl groups by the carboxymethyl groups. Hydrogen bonds sustain the stability of tragacanth gum nanocrystal form, when they are ruptured, it could lead to reducing the crystallinity. As previously reported, the hemiacetalization process led to the formation of a crosslinked three-dimensional network that is partially crystalline or in other terms tends toward an amorphous system. This conclusion was confirmed by the wide-angle peak centered at the 2*θ* value of 20.42 in the XRD pattern of the CMTG-GA polymer (Figure 6(c)). In this context, the XRD pattern of CMTG-GA also illustrates the disappearance of the peaks corresponding to the crystal planes that appeared in the patterns of TG and CMTG. The polymer achieving this partial crystallinity may be due to the acetal bridges between the polysaccharide backbone chains. This phenomenon also corroborates the changes in morphology that will be revealed by SEM.

### 3.5. Morphology Analysis by SEM

**Figure 7:**
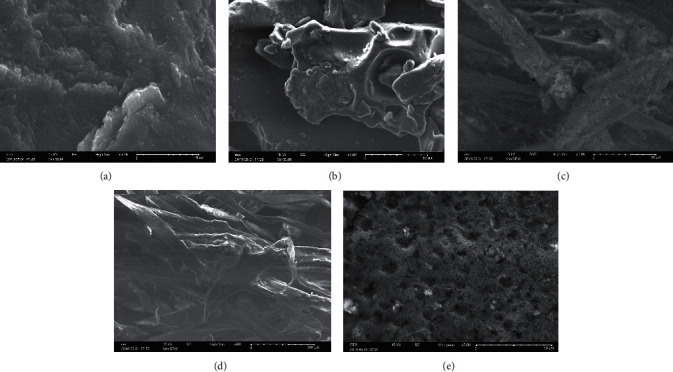
SEM images of TG (a, b) and CMTG-GA (c, e).

SEM was performed to characterize the surface morphology of polymers. The SEM micrographs of TG and CMTG-GA are shown in Figure 7.

As shown in Figures 7(a) and 7(b), TG has only a smooth, homogeneous, and dense surface, whereas a relatively coarse and rough surface of the CMTG-GA superabsorbent polymer is visible in Figures 7(c)–7(e). Some interlaced fibers as a stretched pattern besides a porous surface can also be observed on the surface of CMTG-GA due to the formation of a three-dimensional network by the crosslinking reaction. From a global comparison of the SEM micrographs of the TG and the CMTG-GA, it can be observed that some significant modifications have been made to the surface. In particular, comparing Figures 7(a) and 7(e), which are of the same magnification (10 *μ*m), the appearance of pores on the surface of the superabsorbent polymer can be seen. These modifications confirmed the transformation of the TG structure to CMTG-GA through the crosslinking reaction.

### 3.6. The Effect of Reaction Conditions on the Water Absorbency

#### 3.6.1. The Effect of the Amount of Crosslinking Agent on the Water Absorbency

**Figure 8:**
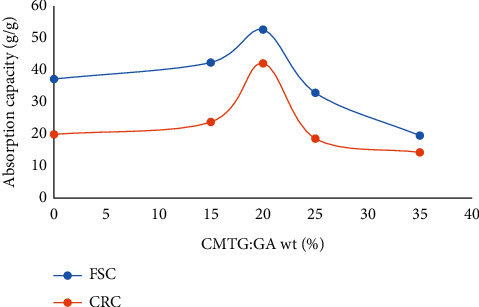
Effect of the crosslinking agent amount on the SAP absorbency.

As the ratio of crosslinking agents has a significant influence on the water uptake of SAP. The hemiacetalization reaction was carried out at different weight ratios of GA to CMTG. The effect of the amount of crosslinking agent (glutaraldehyde) on the water absorption capacity in the synthesis of CMTG-GA was investigated in distilled water to allow the synthesized polymer to achieve maximum performance. The results are presented in Figure 8.

It is observed that FSC and CRC values are marginally improved from 37.2 and 19.9 g/g to 52.63 and 42.1 g/g, respectively, relative to the weight ratio of the crosslinking agent from 0 to 20 wt%, where the maximum values of FSC and CRC are obtained. Therefore, the ratio of GA to CMTG about 20% is the optimized crosslinking density.

This phenomenon is explained by the fact that the optimal degree of crosslinking was achieved, resulting in a greater relaxation of the polymer chains. Eventually, this chain relaxation leads to faster diffusion of water into the polymer matrix [20]. However, in the case where the weight ratios are higher than 20 %, the FSC and CRC values are reduced. This is observed when the weight ratio of the crosslinking agent is 35 wt%, the FSC and CRC values are reduced to 19.52 and 14.27 g/g. Hence, the water absorption of CMTG-GA has been reduced to less than even the initial gum, due to the rigidity of the synthesized structure, which prevents the extension of the polymer chains.

This may be explained by the fact that increasing the crosslinking ratio may increase the probability that hemiacetalization will proceed to the acetal formation. Therefore, crosslinking density is favored by the acetalization process. Acetalization can occur between vicinal diols stereochemically on the same side (cis) of the sugar units on the CMTG backbone, such as L-fucose, L-arabinose, D-galactose, D-xylose, and glutaraldehyde (Figure 1). Vicinal diols are much more reactive with aldehydes than alcohols isolated in an aqueous solution [59]. Hence, acetalization can be considered a competing reaction to hemiacetalization.

To sum up, the reason for this reduction in absorption may be due to the higher amount of crosslinking agent, which in turn promotes the generation of more crosslinking points between the glycosidic chains (bridging points) and subsequently to the increase in crosslinking density. As a result, the network space remaining to contain the water molecules has been reduced to a minimum, as the pore size of the voids available between the network chains has been reduced. Henceforth, the slow diffusion of water molecules into the network causes the absorption performance to decrease.

#### 3.6.2. The Effect of pH on Water Absorbency

**Figure 9:**
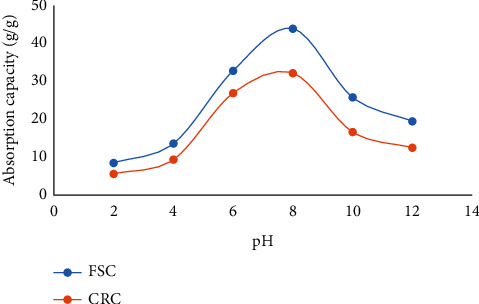
Effect of pH on the SAP absorbency.

In general, the ionic strength of the affected solution has a significant influence on the water absorption of SAP. Therefore, the effect of pH on the water absorption of CMTG-GA under the optimal CMTG:GA ratio has been studied, and the results are presented in Figure 9.

According to the FSC and CRC methods described above, the study was carried out with a light modification in immersion time. The equilibrium swelling ratio was studied in 4 hours at different pH (2.0 and 12.0), allowing the synthesized products to reach maximum absorption. The desired pH stages at 2.0, 4.0, 6.0, 8.0, 10.0, and 12.0 were obtained by diluting hydrochloric acid (pH 1.0) and sodium hydroxide solutions (pH 13.0) with distilled water. Buffer solutions are not suitable for pH adjustment since the ions in the buffer solutions affect the ionic strength of SAP.

The swelling behavior shown by the results obtained indicates that in the range 2.0–8.0, the pH was increased from 8.5 and 5.6 g/g to reach the maximum absorption at 43.9 and 32.14 g/g by FSC and CRC, respectively. However, in the same manner, the values were greatly reduced to 19.47 and 12.5 g/g at pH 12.0.

As known, the immersion of water in the SAP matrix is achieved by the fact that water molecules have diffused into the SAP network through the interaction of water molecules with functional ion groups. Besides, due to the electrostatic repulsion effect between the charges on the polymer chains, the sample dimensions are increased, which explains the observed swelling. In a pH range between 6.0 and 8.0, the carboxylic acid groups were slightly ionized and the repulsion forces between the carboxylate anions increased. On the second side, the hydrogen bonds between the carboxylic acid groups were at the optimum density and water absorption was increased.

By varying the values from pH to acidity, water absorption becomes more negligible. This phenomenon can be explained by two different assumptions. Firstly, the protonation of the carboxylate anions into the carboxylic acid form applies a decrease in the repulsive forces between the carboxylate anions as well as an increase in the rigidity of the polymer matrix due to the resulting rise in the density of the hydrogen bonds. Secondly, in a very acidic medium, during absorption, a reversible reaction of hemiacetalization can take place. As a result, the deterioration of hemiacetal crosslinks and then the risk of losing the crosslinked network will be highly probable. The decrease in water absorption in basic media can be explained by the fact that carboxylate anions were formed by ionization of carboxylic groups, and then the electrostatic repulsion in the network declines due to the screening effect of sodium ions. Consequently, as the pH increases, this phenomenon causes the polymer matrix to collapse.

### 3.7. Analysis of the Swelling Properties of Superabsorbent Polymer

**Figure 10:**
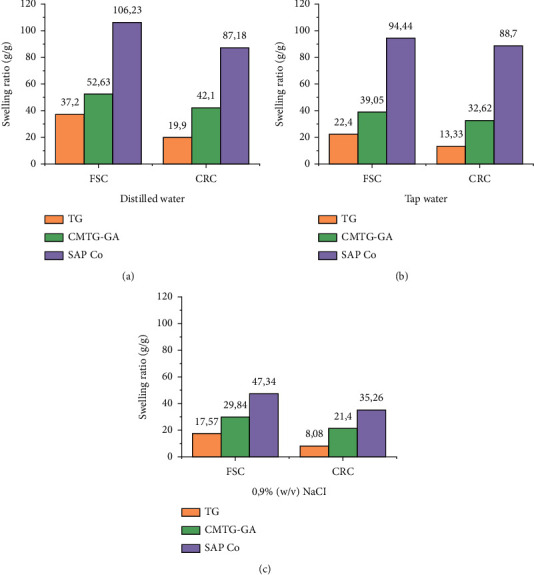
Swelling behavior of TG, CMTG-GA, and the commercial SAP (SAP Co) in distilled water, tap water, and 0.9% (w/v) NaCl.

The swelling study was conducted using the methods reported previously. The results of the absorption and retention capacity of TG, CMTG-GA (optimal CMTG:GA ratio), and commercial SAP in distilled water, tap water, and 0.9% (w/v) NaCl solution are presented in Figure 10.

**Figure 11:**
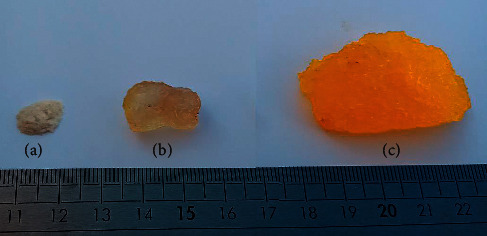
Image of dry TG powder (a), the swollen form of TG (b), and CMTG-GA (c).

The absorption capacity of TG relative to CMTG-GA increased from 17.57, 22.4, and 37.2 g/g to 29.84, 39.05, and 52.63 g/g in saline solution, tap water, and distilled water, respectively. Besides, the retention capacity values are also improved from 8.08, 13.33, and 19.9 g/g to 21.4, 32.62, and 42.1 g/g in saline solution, tap water, and distilled water, respectively. The same observations can be seen in Figure 11.

This improvement in absorbency is related to the transformation of the gum structure into a superabsorbent polymer by the succession of carboxymethylation reactions and crosslinking effect. Despite this development, the performance of commercial SAP was even less observed; 47.34, 94.44, and 106.23 g/g for absorption capacity and 35.26, 68.7, and 87.18 g/g for retention capacity in saline solution, tap water, and distilled water, respectively. As shown in Figure 10, the values of absorption capacity are lower than those of retention capacity, which is explained by the fact that droplets of unbound free water attached to the outer surface of swollen samples have increased mobility compared with bound and half-bound water within the sample matrix and can, therefore, be easily lost during centrifugation [60].

SAPs have several application fields that are exposed to different types of water, namely saline solution, tap water, and distilled water. As shown in Figure 10, the absorbency decreased as the ion content of the solution tester increased. A higher concentration of sodium ions in the saline solution would disturb the hypotonic equilibrium by causing a strong screening effect of the additional Na^+^ ions, consequently decreasing the osmotic pressure difference between the SAP matrix and the external solution, as a consequence of a decrease in absorbency results. Furthermore, the abundant sodium ion in high-concentration saline solution, let alone in tap and distilled water, can permeate into the swollen SAP, reducing the effectiveness of the carboxylate carbonyl ion, decreasing hydrophilicity, and repelling hydrophilic groups [61]. Thus, absorption has to be measured under different conditions to evaluate the swelling properties.

On the other hand, the commercial performance of SAP is relatively higher than that of TG and CMTG-GA in all solutions, as shown in Figure 10. This may be due to the nature of SAP. In contrast to CMTG-GA, which is an almost entirely natural SAP, a commercial SAP is a synthetic polymer, mostly of the polyacrylate and polyacrylamide type, obtained by free radical polymerization of acrylic acid and acrylamide, which are considered to be the best performing SAPs. Moreover, the amount of water absorbed or retained by the polymer network varies as the nature of the polysaccharide and the composition of the polymer networks change [20].

## 4. Conclusions

Tragacanth gum (TG) is a natural gum and a highly complex heterogeneous anionic polysaccharide of high molecular weight. Because of their high biocompatibility and biodegradability, their hydrogels have been extensively investigated. The purpose of this synthesis is to increase the swelling capacity of TG in order to convert it into a superabsorbent polymer. CMTG-GA has been synthesized by crosslinking CMTG polysaccharide backbones previously prepared by carboxymethylation of TG using GA as the crosslinking agent.

According to the experimental results, FTIR analysis has emphasized the confirmation of the formation of the crosslinked polymer. The thermal properties of TG, CMTG, and CMTG-GA are strongly influenced by the crosslinking of the polymer chains. The TGA has shown better thermal stability of the crosslinked product compared with CMTG and pure tragacanth gum. XRD confirms the three-dimensional network formed by the crosslinking reaction, which was demonstrated by the accumulated partial crystallinity; whereas the smooth and homogeneous surface morphology of the TG, as well as the coarse and rough structure of the synthesized material, has been clearly visible on the micrographs performed by SEM. Using FSC and CRC techniques, the swelling studies carried out in distilled water, tap water, and also in a 0.9 wt% saline solution have shown that better swelling is observed when the ratio of GA to CMTG was 20 wt% in all media compared with pure gum. The pH has also affected water absorption. At pH 8.0, the maximum absorption has been 43.9 and 32.14 g/g by FSC and CRC, respectively. By comparing the performance of the synthesized product to commercial SAP, the results for the commercial SAP have been even higher, which is attributed to the nature of the SAP, including the strong performance of acrylic-based SAPs from petroleum derivatives. It is concluded from the previous discussion that the preparation of CMTG-GA has enhanced the swelling capabilities of tragacanth gum, making it an alternative to petroleum-based superabsorbent polymers in a variety of applications. Finally, for more extensive research on the application of the SAP, the gel fraction, mechanical properties, ionic strength, and swelling behavior of CMTG-GA in the soil can be studied to better assess the performance and limitations of SAPs.

## Figures and Tables

**Table 1:** Weight ratios of GA to CMTG (wt %).

Sample	Crosslinking agent ratio (wt%)	GA (g)
TG	0	0
CMTG-GA 1	15	0.09
CMTG-GA 2	20	0.13
CMTG-GA 3	25	0.17
CMTG-GA 4	35	0.27

## Data Availability

The data used to support the findings of this study are included within the article.

## Abbreviation

CMTG: Carboxymethyl tragacanth gum
CMTG-GA: Carboxymethyl tragacanth-glutaraldehyde
CRC: Centrifuge retention capacity
FTIR: Fourier transform infrared
FSC: Free swelling capacity
GA: Glutaraldehyde
REACH: Registration, evaluation, authorisation, and restriction of chemicals;
SAP: Superabsorbent polymer
SEM: Scanning electronic microscopy
TG: Tragacanth gum
TGA: Thermogravimetric analysis
XRD: X-ray diffraction
wt%: Percentage by weight.
